# Mucormycotic Osteomyelitis Involving the Maxilla: A Rare Case Report and Review of the Literature

**DOI:** 10.1155/2019/8459296

**Published:** 2019-01-22

**Authors:** Rajesh Arani, S. N. H. Afsar Shareef, H. M. Khuthija Khanam

**Affiliations:** ^1^Dept. of Oral Pathology and Microbiology, G.Pulla Reddy Dental College and Hospital, Kurnool, Andhra Pradesh, India; ^2^Consultant Prosthodontist and Implantologist, Continental Hospitals, Hyderabad, India; ^3^Department of Restorative and Prosthetic Dental Sciences, Dar Al Uloom University, Riyadh, Saudi Arabia

## Abstract

Osteomyelitis is an inflammatory process of bone and marrow contents. These changes in bone are primarily seen in soft tissue followed by calcified tissue. It is an opportunistic infection due to the complication of some other conditions rendering the host susceptible to disease. Consequences of this infection range from draining tract to malignant transformation. Various etiological factors are involved in origin of the disease; among them, fungal origin is rare. Specific feature in fungal osteomyelitis is the involvement of maxillary sinus with a complaint of sinusitis associated with diabetes mellitus. Here, we discuss a case of osteomyelitis with fungal infection involving the maxilla. The patient is under medication for the past five years due to diabetes.

## 1. Introduction

Maxilla is one of the primary bones of the face and forms the upper jaw. It is a vital structure of the viscerocranium and is involved in the formation of the palate, nose, and orbit. The alveolar process of maxilla holds the upper teeth and plays an important role in mastication and speech. Maxillary necrosis occurs rarely compared to the mandible due to its high vascular supply [1]. If maxillary necrosis occurs, it may be due to bacterial infections such as osteomyelitis, viral infections such as herpes zoster, or fungal infections such as mucormycosis or may be due to trauma, radiation, etc. [2]. However, long-term use of antibiotics or corticosteroids may also result in an opportunistic fulminant fungal infection, mucormycosis, which mainly affects the immunocompromised patients. These fungi are commonly seen in many individuals, but the symptoms were associated with a poor immune system. Mucormycosis is a life-threatening infection that occurs commonly in immunocompromised patients, because of diabetic ketoacidosis, neutropenia, organ transplantation, and increased serum iron levels [3].

It is an angioinvasive infection due to filamentous fungi of the class Zygomycetes and the order Mucorales [4] and was reported with an annual incidence of 0.07% and 0.29% in the patients who underwent solid organ transplant and hematopoietic cell transplants [5].

The occurrence of mucormycosis has raised in developing countries including India. Three consecutive case series on mucormycosis have been reported from a single tertiary centre in India: from 1992 to 1999, i.e., for a period of 10 years, 129 cases have been reported; from 2000 to 2004, i.e., for a period of 5 years, 178 cases have been reported; and from 2006 to 2007, in an 18-month period, 75 cases have been reported [6].

The increasing incidence of mucormycosis in India has been attributed primarily to a continued increase in patient population with uncontrolled diabetes. In addition to this, the environmental factors such as humid climate and high temperatures in most parts of India provide an optimum setup for these fungi to survive and perhaps contribute to the disease prevalence. Reviewing the Indian literature over the past five decades (1960–2012) showed the prevalence rate of mucormycosis as 0.14 cases per 1000 population [6].

## 2. Case Report

**Figure 1:**
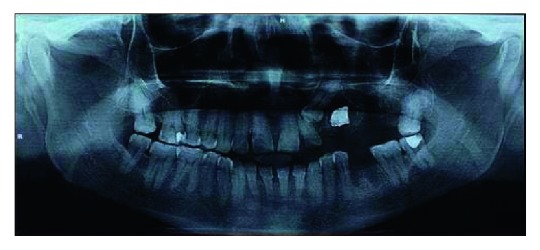
Orthopantomograph.

A 48-year-old male patient visited dental hospital with pain and swelling, along with pus discharge in the left posterior back tooth region of the upper jaw since one week. The patient presented the complaints of nasal regurgitation, cough, and intermittent fever since one week. The patient underwent extraction of 26, twenty days back. Pain was mild, continuous, and localised which aggrevated on talking and relieved on medication. Intraoral examination showed opening along the alveolar ridge extending deep into the cortex in relation to 26 (Figure 1). The OPG revealed radiolucency extending from the alveolar ridge to maxillary sinus, breaking the floor of the sinus in relation to 26. The patient is diabetic, and he is under medication for the last five years.

**Figure 2:**
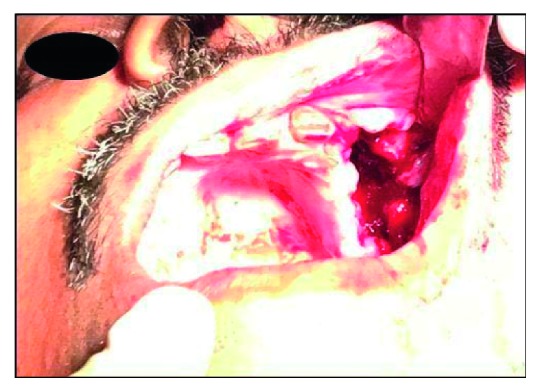
Clinical picture during excisional biopsy.

**Figure 3:**
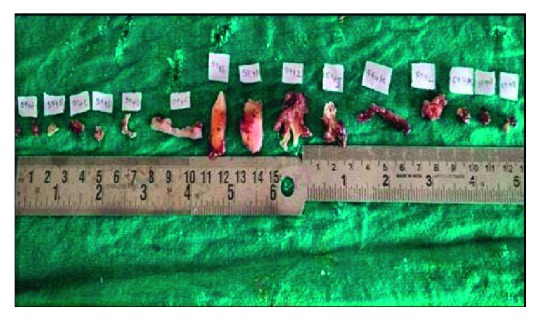
Tissue during grossing.

The patient was advised for excisional biopsy, and the tissue specimen was sent for microscopic examination (Figure 2). The oroantral opening was closed surgically (Figure 3). The biopsy specimen showed consists of soft tissues, bone bits, and extracted teeth. The soft tissue is whitish grey in colour, firm in consistency, and irregular in shape.

**Figure 4:**
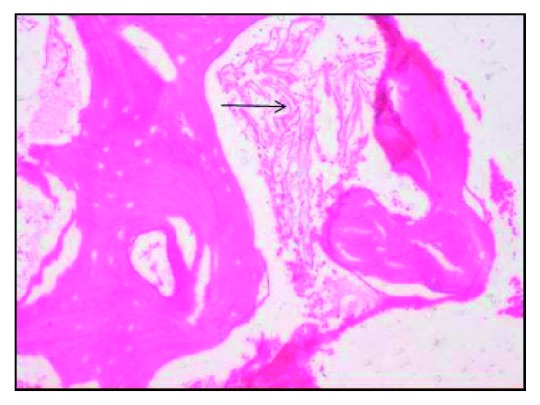
H&E-stained decalcified section showing hyphae under 20x.

**Figure 5:**
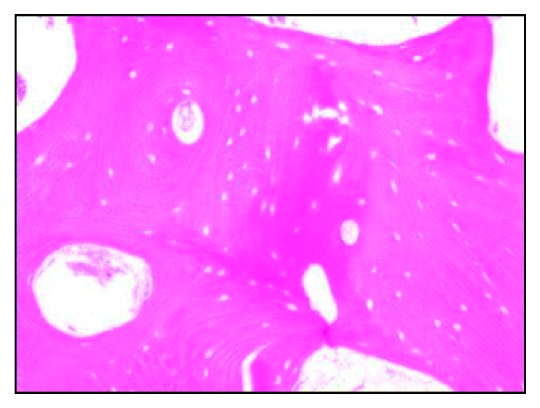
H&E-stained decalcified section with empty lacunae under 20x.

**Figure 6:**
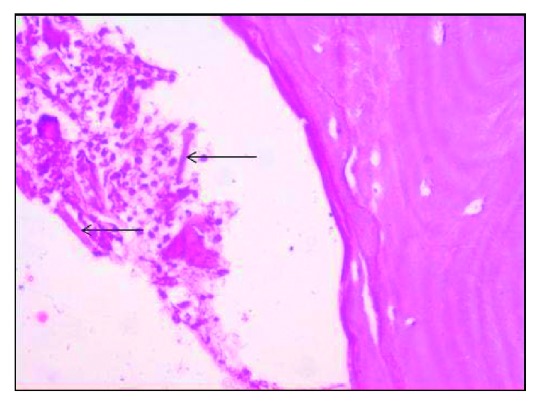
PAS-stained decalcified section showing fungal hyphae in magenta colour under 20x.

Microscopic examination showed parakeratinised stratified squamous epithelium in association with loosely arranged collagen fibrous connective tissue. Numerous hyphae were seen which were broad, septate, branched, and scattered all over the connective tissue and admixed with chronic inflammatory cells. Figures 4 and 5 show the decalcified section of bony trabeculae with empty lacunae without osteoblastic rimming interspersed with little fibrous connective tissue and the fungal hyphae. For confirming the fungal hyphae, the PAS staining was done, which also showed the magenta-coloured hyphae in the PAS staining (Figure 6). With respect to the microscopic features seen, the disease is diagnosed as mucormycotic osteomyelitis.

## 3. Discussion

The term osteomyelitis is derived from the Greek word osteon meaning bone, myelo meaning marrow, and itis meaning inflammation. Nelaton coined the term “Osteomyelitis” in 1844 [7]. Osteomyelitis is inflammation of the bone that begins in the medullary cavity and ends in the periosteum involving the haversian system [8]. Various factors are involved in the development of the disease such as trauma, surgical therapy, bacteremia, fungal infection, and systemic diseases that decrease host defence mechanism such as diabetes, malignancy, anemia, radiation, malnutrition, osteoporosis, osteopetrosis and Paget's disease [9].

Contiguous spread of infection from surrounding soft tissue and bones due to hematogenous seeding or direct inoculation of microbes into bone results in the disease origin [10]. In all these conditions, the vascular supply is decreased, thereby predisposing the infection. Entry of microbes into cancellous bone causes the compression of blood vessels preceeded by the inflammation and edema of marrow [9]. Severe compression of vascularity leads to ischemia and necrosis of bone. Immobile and stagnant blood leads to nidus for development of infection [11]. Osteomyelitis is commonly seen in the males (80.36%) than in females (19.64%), with a peak incidence in 30–39 years of age [12].

Osteomyelitis involving the facial bones is rare, and involvement of maxilla is less common than that of mandible due to high vascularity [1]. Even though broad-spectrum antibiotics decreased the prevalence of the disease, it still remains as a challenging entity in developing countries and low socioeconomic groups [9]. Osteomyelitis occurring due to fungal infection is rare and occurs in an indolent manner [13].

Urs et al., in their prospective study which was undertaken from 2011 to 2013 December, could find only five cases which showed the characteristics of fungal osteomyelitis. All those five cases were primarily intraosseous and have shown the radiographic changes in bone. Out of these five cases, three cases involved maxilla and two patients of these three cases were presented with a history of uncontrolled diabetes [1].

Niranjan et al., in their ten-year study (from January 2005 to December 2015) which was designed to evaluate the prevalence of fungal osteomyelitis of the jaws associated with diabetes mellitus, reported that 52% of all the osteomyelitis cases were that of fungal osteomyelitis, whereas 48% belonged to the nonfungal category. In the same study, they also reported that fungal osteomyelitis was frequently found in the individuals above 40 years of age and is more common in males when compared to females [14]. Maxilla is the most common jaw bone being affected by fungal osteomyelitis and is more commonly associated with diabetes mellitus [14] (Table 1).

**Figure 7:**
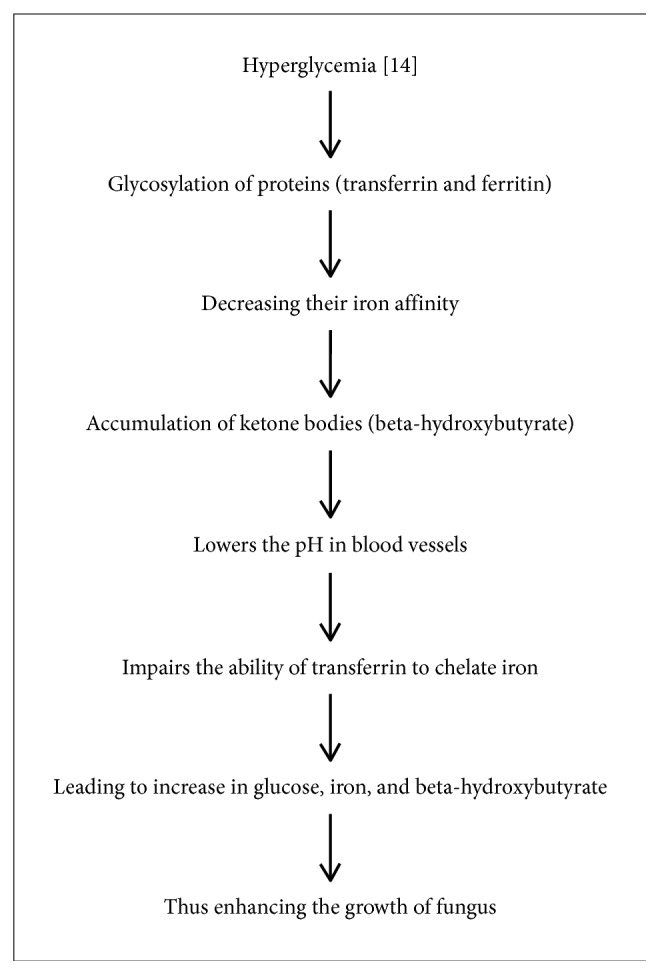


In 2016, Siddanagouda Biradar et al. reported a case of mucormycosis in a diabetic patient. His urine analysis revealed 1.5% glucose & ketone bodies and albumin of more than 2% [15]. Maxillary to mandibular ratio in Peravali et al.'s study is 1.07 : 1, and according to Koorbosch et al., ratio is 1.6 : 5, and according to Rangne and Ruud, ratio is 1 : 6 [16]. This is due to the rich vascularity of maxilla [1]. In maxillary osteomyelitis, diabetes mellitus is usually a propagating factor. In diabetic patients, the presence of ketone bodies favour the suitable environment for the growth of fungus. Ketoreductase is the enzyme produced by the fungus that acts on the ketone bodies [1]. Vijaya bala et al. also reported a case of mucormycosis in a diabetic ketoacidosis patient [17] (Figure 7).

Coming to our case, i.e., the patient with maxillary fungal osteomyelitis, he is under medication for diabetes since five years. Osteomyelitis in tooth-bearing bone usually occurs due to polymicrobial odontogenic bacteria such as bacteroides, peptostreptococcus, and microaerophilic streptococcus along with the opportunistic pathogens, whereas in long bones, *Staphylococcus aureus* is the most causative organism. Osteomyelitis due to fungal organisms is rare, but it is seen in immunocompromised patients [9]. In our case, the microscopic examination showed the fungal hyphae which are broad and septate.

Fungal organisms usually causing osteomyelitis are *Candida parapsilosis* and *Aspergillus*. These organisms are from original infection that has not been treated properly, commonly from dental extraction [1]. In our case also, the patient gives the history of 26 extraction. Among fungal osteomyelitis, Candida is the most commonly encountered followed by aspergillosis and mucormycosis [1, 18]. Candidal osteomyelitis occurs as simultaneous or late manifestation of hematogenous-disseminated candidiasis. *Aspergillus* is the second most organism that are usually seen with osteomyelitis [19]. *Aspergillus* is a saprophyte that causes the opportunistic infection in patients with immunosuppression, chemotherapy, transplantation, uncontrolled diabetes and Intravenous drug abusers with altered immune defence mechanism [8].

Phagocytes containing the NADPH oxidase (nicotinamide adenine dinucleotide phosphate hydrogen) produce the defense mechanism against the *Aspergillus*. Activated NADPH oxidase leads to production of reactive oxygen metabolites causing the antimicrobial activity [20]. In neutrophils, this antimicrobial activity is linked with activation of intracellular proteases [21]. In the study of Gabrielli et al., from 1936 to 2013, *Aspergillus* causing osteomyelitis is reported in 310 patients [22]. Mucormycosis usually seen in immunocompromised patients is an opportunistic fulminant fungal infection [23]. It is also frequently seen in the diabetic patients as the ketone bodies favour the favourable environment for the growth of the organism [1] (Table 2).

Trifungal osteomyelitis presence of three types of fungus, *Candida*, *Aspergillus*, Mucormycosis is rare condition, only two cases were reported [1]. Clinical features of fungal osteomyelitis is similar to bacterial osteomyelitis, exposed bone and pain with varying intensity. Fungal osteomyelitis in the form of cold abscess is rare [8]. Fungal osteomyelitis is more invasive than the bacterial, if not diagnosed and treated earlier [25].

As per the guidelines from the 3rd European Conference on Infections in Leukemia (ECIL 3), the firstline treatment of mucormycosis is as follows [26]:

1. AmB deoxycholate
2. Liposomal AmB 5–10 mg/Kg
3. Posaconazole 400 mg bid
4. Combination therapy
5. Control of the underlying condition
6. Surgery
7. Hyperbaric oxygen therapy

2nd line of treatment is as follows:

1. Posaconazole 400 mg bid
2. Combination lipid AmB and caspofungin
3. Combination lipid AmB and posaconazole
4. Combination deferasirox not recommended
5. Maintenance therapy posaconazole

Chronic osteomyelitis often presents a chronic draining fistula although rest of the features are similar to acute counterparts.

### 3.1. How to Diagnose the Disease?

The diagnosis of mucormycosis is challenging, and it should be treated as early as possible, otherwise it may be fatal. There are no recommendation to guide the diagnosis and management of mucormycosis in spite of the infection morbidity and mortality of the affected patient. However, the American Infectious Disease Society has presented certain guidelines to be used on evidence criteria [26].

The guidelines in brief are as follows:

1. The diagnosis of mucormycosis relays on histology and/or detection of the organism by culture from the involved sites with identification of the isolate of the species level
2. Antifungal chemotherapy should be able to control the underlying predisposing condition after surgical debridement

Computed tomography and magnetic resonance imaging techniques were used as early diagnostic tools. Bone scintigraphy is more accurate compared to CT scan because bone erosion and remodelling in CT may be confused with osteomyelitis [27]. Hyphae were microscopically identified with stains like H&E (hematoxyline and eosin), PAS (per iodic schiff), and GMS (Grocott's methanamine silver), and in particular, the type of hyphae whether septate (or) nonseptate is seen by GMS staining [1]. Identification of hyphae was done through histological sections but the exact species were identified only through culturing. Coming to our case, the microscopic examination revealed the bony trabeculae consisting of empty lacunae intermixed with broad and septate hyphae, branching greater than 90°. These features are suggestive of mucormycotic osteomyelitis. Further, the specimen is stained with the PAS stain which shows the fungal hyphae in magenta colour.

## 4. Treatment

As said earlier, fungal osteomyelitis is more invasive than bacterial, if not diagnosed and treated earlier. The treatment given to our patient is as follows:

1. Local debridement of necrotic tissue, eradicating the fungal pathogen under local anesthesia
2. Antifungal medication
    Posaconazole 300 mg tablets twice daily (1 bid) for 15 days
    Hydrogen peroxide mouthwash 3 times a day for 15 days
    A to Z (multivitamin) capsule for 15 days

## 5. Follow-Up

The patient has been asked to come for a checkup after 3 days, 1 week, and fortnight (15 days).

## 6. Conclusion

Osteomyelitis is the oldest disease known from the literature. But, fungal osteomyelitis is a rare entity, usually seen in patients with diabetes and immunosuppression. Care should be taken in accurate diagnosis through investigations such as histological and radiological. These investigations confirm whether the osteomyelitis is of fungal origin (or) not. However, identification of fungal species can be done only through the culture. Accurate antifungal treatment is needed for the fungal osteomyelitis. Complications are sinus draining to bony destruction.

## Figures and Tables

**Table 1:** Prevalence of fungal osteomyelitis as per Niranjan et al.

52% fungal	48% nonfungal
69% males	30.77% females
80.76% maxilla	19.24% mandible
61.53% diabetic	38.47% nondiabetic

**Table 2:** The differences between *Candida*, *Aspergillus*, and mucormycosis-causing fungi [1, 24].

	*Candida*	*Aspergillus*	Mucormycosis-causing fungi
Hyphae	3–5 micrometre diameter	3–6 micrometre diameter	6–20 micrometre diameter
	—	Septate	Septate and broad
	Appears yeast-like		
	Intermixed with pseudohyphae (filaments)		
Branching	—	Dichotomous	Greater than 90°
		Between 45°–90°	
